# Supplementary material for: Patterns of Intron Gain and Loss in Fungi
Source: PLoS Biol. 2004 Nov 30;2(12):e422. doi: 10.1371/journal.pbio.0020422 (PMC532390; doi:10.1371/journal.pbio.0020422)
Supplement: Table S1 — Also available at http://genes.mit.edu/NielsenEtAl/. (4.3 MB ZIP). [file pbio.0020422.st001.zip › NielsenEtAl/html/313.html]

AN8291.1.NCU04652.1.MG07481.1.FG07432.1


```
 CLUSTAL W (1.82) Multiple Sequence Alignments - Introns Inserted


Sequence 1: NCU04652.1	960 aa
Sequence 2: MG07481.1	994 aa
Sequence 3: FG07432.1	940 aa
Sequence 4: AN8291.1	962 aa
Alignment Length: 1095 aa
Number Identitical Residues: 313 aa
Alignment Score (without introns) 16696


MG07481.1 	-~-------------~-----~-----------------~-----------~--------
NCU04652.1	-~-------------~-----~-----------------~-----------~--------
FG07432.1 	-~-------------~-----~-----------------~-----------~--------
AN8291.1  	M0TNQVDGVLRQNIV0LLVKL1VKRLPARSSLTSNLAQN0VREVVVRPDKS2LRECRAEI
          	  :.. ..   ..      .   .  .: :: ::. :..   .    ...:   .. :. 

MG07481.1 	----MD~LKATALLKLVYLEMMGHDMSWASFHVLEVMSSPKIHQKRLGYLGAVQTFRPDT
NCU04652.1	--MRKD~VKATALLKIIYLEMNGHDMSWASFHVLEVMSSPKYHQKRVGYLGAVQSFKTDT
FG07432.1 	------~------------------MSWASFHVLEVMSSPKYHQKRVGYLGAVQSFRPDT
AN8291.1  	RSQDMD1KKATALLKLAYLEMFGYDMSWASFNVLEVMSSSKFLQKRVGYLGALQSFRPET
          	 :   .  .:::  .    .  . .******:*******.*  ***:*****:*:*:.:*

MG07481.1 	EVLMLATNLLKK~DLMATAPNTIGLPIITLPHIITPSLALSVLADLLPRLSHSHASIRKK
NCU04652.1	EVLMLATNLLKK~DLSASSPIVISLPIAALPHIITPSLALSVLADLLPRLSHSHSAIRKK
FG07432.1 	EVLMLATNLLKK0DLGTTTPTVISLPIATLPHVITPSLALSTLQDLLPRLSHSHSNIRKK
AN8291.1  	EVLMLATNLLKK0DMVCSNLQIISLPLNTLPNIITPSLAMSLLPDVLSRISHSSPSIRKK
          	************ *:  :    *.**: :**::******:* * *:*.*:*** . ****

MG07481.1 	TIVTLYRLALVYPETLRAAWPKIKDRLMDKDEDPSVTAAIVNVVCELGWRRPHDFLPLAP
NCU04652.1	TIVTLYRLALVYPETLRAAWPKIKERLMDKHEDPSVTAAIVNVVCELGWRRPQDFLPLAP
FG07432.1 	TLVTLYRLALVYPEALRAAWPKIKERLMDPDEDPSVTAAIVNVVCELGWRRPNDFLPLAP
AN8291.1  	AVVCLYRLALVYPEALRLAWPRLKDRLMDETEDSSVTTAVLNVVCELGWRRPHDFLPLAP
          	::* **********:** ***::*:****  **.***:*::***********:*******

MG07481.1 	RLFELLVEGGNNWMAIKLIKL~FATLTPLEPRLIRKLLPPLTDIIRTTPAMSLLYECING
NCU04652.1	RLFELLVDGGNNWMAIKLIKL0FATLTPLEPRLVRKLLPPLTDLIRTTPAMSLLYECING
FG07432.1 	RLFELLVDGGNNWMAIKLIKL0FATLTPLEPRLVRKLLPPLTNIIRTTPAMSLLYECING
AN8291.1  	RFFELLVEGGNNWMSIKIIKL0FATLTPLEPRLVRKLIRPLINIVQTTTAMSLLYECING
          	*:*****:******:**:*** ***********:***: ** ::::**.***********

MG07481.1 	IIQGGILGSPDDISGTEEIATLIVSKLRGMIMIDGDPNL1KYVALLA~FNKIVTTHPFLV
NCU04652.1	IIQGGILGDSEDD-GREEIASLCVSKLRGMITFNGDAN-~------S1FNRIVVTHPFLV
FG07432.1 	IIQGGILGNSDDS-GTDEIATLCVNKLRGMIMIDGDPN-~-------~-----------L
AN8291.1  	IIQGGILEGEGALEDSHEIADLCVSKLRGMVVTDFDPN-~-------~------------
          	******* .    .. .*** * *.*****:  : *.*                      

MG07481.1 	~AEQEDVILECID~SPDITIRIKALDLVQGMVSADNLESIVSRLMRQLKVASEGDRQPKQ
NCU04652.1	~AQQEDVIMECID~SEDITIRIKALDLVQGMVSSDNLVSIVSRLMRQLKTSTNPNNDQLD
FG07432.1 	1SQQEDVILECID~SPDITIRIQALDLVQGMVTGDNLMSIVSRLMKQLKLSMPSRDKSQP
AN8291.1  	~-----------Q1DADISIRLQALELAARMVTGDTLQPVVERLIGQLEEPQHTFPKG-D
          	            : . **:**::**:*.  **:.*.* .:*.**: **: .     .   

MG07481.1 	TQTDSDVEDDGLSNINTKSRATNSPPPLPEEYRIDVIGRIIHVCSLDNYNNLLDFDWYID
NCU04652.1	DLSTDSSEEMGAE---RRSRKRDEAPPLPEDYTIDVIGRILKMCSQNNYSSVVDFDWYID
FG07432.1 	GTPPNDFNESEDEYAESTPKPKSESIPLPDDYRIDVIGRILGMCSKDNYSSVLDFDWYID
AN8291.1  	ASDAGDVAVLANHVGRRELGKHPAYFPISDEYRVEILHRVLDICSHDNYSRLTDFEWYVS
          	    ..                    *:.::* :::: *:: :** :**. : **:**:.

MG07481.1 	ILTQLVRMAPVARRKEDEDDSSVTTTKTRATDITERIGNELRSIAVKVQA~IRGSAVRAA
NCU04652.1	VLTQLIRIAPASR-KELDSDGMALGPKSTTADISESIGNELRNVAVKVKA0L--------
FG07432.1 	VLTQLVRMAPAPR-KVDDDSG---PTDKARANVSEKIGDELRNVAVKVRV~MRSTAVRAA
AN8291.1  	VLIRLVKHLPTRVEEHTVSQG---FESSSRDDAASRIGLEMRNIAVRVKN~VRMEATRAA
          	:* :*::  *.   :   ...     ..   : :. ** *:*.:**:*:  :   :  ::

MG07481.1 	ESIISGLGSESST------TTKVVGPVSWILGEYASFLTHPEDTMNAILQALAKTPYPEV
NCU04652.1	---------------------------------------------------IPRIRPSEV
FG07432.1 	ETILSQLNTDTPPG--YSITSGALKSVAWIMGEYASQLAVTDEGLNGLLQLIPRTNTPEV
AN8291.1  	EFLLLVDNRQSVFANVSLAYNGVLGPLAWVVGEYAEYLSSPGPMLQSLIDVSTTSLSGRA
          	.      . .:  ..     .    . :   .. :.  : .    ..  .  .     ..

MG07481.1 	AAISMHALMKIFALVVGDERSQWTAERKAKISLLMTRLLHTLEPFPSHPNLEVQERAVQF
NCU04652.1	LATCLQASMKLFALIAGNDQTYWTAERKSMVSLLMARVIHTYEPLVLHPSLEVQERAVEF
FG07432.1 	LTTTLQAVTKVFATIVGDESEPWTAERKSRISLLMARIIHVFEPLALHPSLEVQERAVEF
AN8291.1  	LVLFVQAVPKVLARIVHDYMGTWDVRQRSEVSLFLARIVEFYEGLATHPDLEVQERAIEF
          	 .  ::*  *::* :. :    * ..::: :**:::*::.  * :  **.*******::*

MG07481.1 	TELLKLTAEAVSGQEASTDEVYQDPPLLLTQALPSLFTGWELNSVAFGAQNNVPLPEGLD
NCU04652.1	TELLKLTAEAASAQASSTDELEQDPPLLLTQAIPSLFQGWELNSVAVGAQLNVPMPEGLD
FG07432.1 	TELLKLTAEAASSQPSSTDDVDQDPPLLLTQAIPSLFNGWELNSVSKDAQLNVPVPSGLD
AN8291.1  	LEIMRLAADVMQ-----TSTQQAQVPYLLSSIIPSLFYGLELNPVAPNAQGKVPLPDQLC
          	 *:::*:*:. .     *.    : * **:. :**** * ***.*: .** :**:*. * 

MG07481.1 	LDQPINPNLERLLAEAHMLNLPVDEDDEFEIYYHQKPVATSISSSEPAIKRLADAPDEIP
NCU04652.1	LDEPIHPNLSSLLAQADSLMLPIQEDDEFSAYYNEKAAPTSISS-EPAINRLRDAPETTS
FG07432.1 	LDEPIHANLAKLLSEADSITLATDESDDFETYYHQRPPPTSIESSAPAISRIAEPKEDYV
AN8291.1  	LNVPFNDNVRKLFESYQDG-FYAQPYDFYRVGVTQLPHKQ-----QGGFNTIEIQPN---
          	*: *:: *:  *: . .   :  :  * :     : .          .:. :    :   

MG07481.1 	NSYQNAGEESYLDPDILA--RRKAERMDKNKDDPFYIHNTSSG--ISRTSTPIHNILKRE
NCU04652.1	GSYQQATEDSYLDADIIA--RRKAERAERNRDDPFYIGDLGSPSRSQGTSTPIHNILQNE
FG07432.1 	SSYQQADEETYLDADIVA--RRKAERVERNRDDPFYIPSNETP----RTSTPIHNILQSS
AN8291.1  	FSYQDPD---ILDEALAASLRQKLEWKQRNREDPFYIGADERN----DATAPTERPFGSV
          	 ***:.     **  : *: *:* *  ::*::*****           :::* .. :   

MG07481.1 	NGSDLDIDSIPIMALDLSKVDDAAPQKPPAPLPR---PKSRQRVVVAADETLAGSGRSTP
NCU04652.1	NGPDLDIDSIPIMKLDLGAISSSGVGGAPGTPQRSKAPKSRPQIVVAADETLVGSRPSTP
FG07432.1 	NGPDLDIDSIPIMQLDLSRAETPAAQ-PPRPQPK-----PRQKVVIAADETLEGSASGGL
AN8291.1  	HSEALDVDSIPIIDLKISGLGDESTPKARSYDSQGKLAGQPKKYEVIADEVLD-------
          	:.  **:*****: *.:.     .   .     :.. .    :  : ***.*        

MG07481.1 	GNYDSENTNSDGGRKTAAVKSKQHKRPKNSLLQVDSSHIGALSLHGRADEEAVSSVDPEL
NCU04652.1	R--TGSQHDSDADPSSASAKSRAARKLKQSLLQVDSSHLRNLSLEASHSEDSGGGALPHE
FG07432.1 	R-----SYDSENNSDSLSKPRVFKTKIKQGLLGVDSSGIGSFSLEGQPS----TGLDYEQ
AN8291.1  	-------LEETADFSSPDEP----VKAKRALLQVDSSGLKDLTLG----DDGLAHANGVP
          	        :.  . .:         : *..** **** :  ::*     ...        

MG07481.1 	QKRE-EAEMAAAMKEVERLRLEMQRANERIQVAQGVPAEGVAVVQKKKK-------KKKA
NCU04652.1	KLAETDAEMAKAMKEVQRLRLEMQRANERIHVAQGVPTEGVVVKSKKKKEGKKAKSKSKS
FG07432.1 	QQRE-DVEMQQAMKEVERLRLEMQRANERIDVAQGVDVEGTVVKKKKKK------PKKDV
AN8291.1  	GKSEDDAEMATAMKEIEKIRLKMQRASERVELEG-APSDGMVVKKRRKP-----------
          	   * :.**  ****::::**:****.**:.:   .  :* .* .::*            

MG07481.1 	VPDGE---DGDVASSVAKPKKKKKKPPVALGG-EGDTGAAA--AEGEVVAKPKKK-----
NCU04652.1	EEDGAGAGEDEKEKKVVKKKKKKAETEVAEGGGEGAPVGGE--GEGEAVITVKKKEKEKV
FG07432.1 	TTEGA---DGEEVKPKKKKKKTPRAVALEEGGDDSSARGVESAASGADVAVAKKK-----
AN8291.1  	----------------KKTSHNKTRKGYSLQGENSQSEHGK---SSSTVHKRTRK-----
          	                 * .:.         * :. .       ..  *   .:*     

MG07481.1 	-KKKP---------AAEGEGTAGDSGLI
NCU04652.1	KKKKPKRIIQLGDEDAGGTGAGAEAGEP
FG07432.1 	-KKKK----------APRAAEIQDE---
AN8291.1  	-RKGD----------AERLG--------
          	 :*            *   .
```
